# Crystal Structures of Two Isozymes of Citrate Synthase from* Sulfolobus tokodaii* Strain 7

**DOI:** 10.1155/2016/7560919

**Published:** 2016-08-30

**Authors:** Midori Murakami, Tsutomu Kouyama

**Affiliations:** Department of Physics, Graduate School of Science, Nagoya University, Nagoya 464-8602, Japan

## Abstract

Thermoacidophilic archaeon* Sulfolobus tokodaii* strain 7 has two citrate synthase genes (ST1805-CS and ST0587-CS) in the genome with 45% sequence identity. Because they exhibit similar optimal temperatures of catalytic activity and thermal inactivation profiles, we performed structural comparisons between these isozymes to elucidate adaptation mechanisms to high temperatures in thermophilic CSs. The crystal structures of ST1805-CS and ST0587-CS were determined at 2.0 Å and 2.7 Å resolutions, respectively. Structural comparison reveals that both of them are dimeric enzymes composed of two identical subunits, and these dimeric structures are quite similar to those of citrate synthases from archaea and eubacteria. ST0587-CS has, however, 55 ion pairs within whole dimer structure, while having only 36 in ST1805-CS. Although the number and distributions of ion pairs are distinct from each other, intersubunit ion pairs between two domains of each isozyme are identical especially in interterminal region. Because the location and number of ion pairs are in a trend with other CSs from thermophilic microorganisms, the factors responsible for thermal adaptation of ST-CS isozymes are characterized by ion pairs in interterminal region.

## 1. Introduction

Proteins from thermophilic organisms exhibit unique structural properties for high thermal stability. Structural studies have suggested that various factors are responsible for the thermal adaptation mechanism, for instance, a large number of ion pairs and ion pair networks, tighter intersubunit contacts, reduction of flexibility of the N- and C-terminal polypeptides, and reduction in both the number and the total volume of internal cavities [1–5].

Citrate synthase (CS, EC 2.3.3.1), an enzyme involved in the TCA cycle, catalyzes the condensation of oxaloacetate and acetyl-coenzyme-A (CoA) to form citrate and CoA. CS has been isolated from various organisms, including eubacteria and archaebacteria. CSs from eukarya, archaea, and Gram-positive bacteria have dimeric structures under physiological conditions, whereas those from Gram-negative bacteria are isolated as homohexamers with some exceptions [6, 7]. Remington and his colleagues have previously determined the structures of CSs from pig and chicken mitochondria [8, 9]. Dimeric CSs from microorganisms living in a wide temperature range, that is, from psychrophiles to hyperthermophiles, have also been investigated [10–14]. The enzymes consist of predominant *α*-helices. In contrast to pig CS comprising 20 helices (A to T helices), archaeal CS lacks A, H, and T helices and has 16 helices in total and forms homodimers, in which each subunit has polypeptide length of approximately 380 residues, 50 residues shorter than eukarya CSs. Each polypeptide comprises two domains, the large domain, which is responsible for dimer formation, and the small domain, which shows a rigid-body rotation relative to the large domain upon binding the substrates in the active site cleft between these two domains. Comparative studies of microbial CS structures are performed intensively and focus on the trends that appear to be associated with the increasing thermostability [5, 10–14]. However, multiple factors seem to be entwined in this thermal stability problem because these microorganisms live not only in various temperatures but also in various environments such as pH and salt strength.

Thermoacidophilic archaeon* Sulfolobus tokodaii* strain 7, which grows optimally at 75°C and pH 3, has two genes encoding two distinct isozymes of hypothetic CS [15]. One of them encoded by an open reading frame (ORF) ST1805 is termed ST1805-CS, and the other, encoded by the ORF ST0587, is termed ST0587-CS. These two isozymes contain the consensus sequence of citrate synthase. Their amino acid sequences show 45% identity despite being derived from the same organism, implying that the two isomers of ST-CS have evolved branch lines in a phylogenetic tree.

We confirmed these two isozymes to function in high temperatures as CS and determined the crystal structures of them at resolutions of 2.0 Å and 2.7 Å, respectively. The crystal structures of ST-CSs allow us to make detailed comparison of the enzyme structures in relation to their thermostability and thermoactivity and understand common factors for adaptation mechanisms to high temperatures in ST-CSs and CSs from other sources.

## 2. Materials and Methods

### 2.1. Purification of the Recombinant Proteins

*E. coli* (Rosetta-gami DE3) transformed with plasmids pST1805 and pST0587 were grown at 37°C in LBL (Luria-Bertani-Lennox) medium containing 100 *μ*g/mL carbenicillin and 20 *μ*g/mL chloramphenicol. The cells were harvested by centrifugation at 4600 ×g for 10 min, washed with 50 mM NaCl, and resuspend in a Tris buffer (20 mM Tris-HCl (pH 8.5), 1 mM EDTA, 1 mM 2-mercaptoethanol, and 1 mM PMSF). The harvested cells were disrupted by sonication using an ultrasonic homogenizer (150 W). Cell debris and large particles were removed by centrifugation at 20,000 ×g for 20 min. The supernatant fraction was incubated at 90°C for 10 min for denaturation of intrinsic proteins of* E. coli* and their denatured proteins were removed by centrifugation at 20,000 ×g for 30 min at 4°C. The supernatant was then applied to a 25 mL affinity column (Red-TOYOPEARL) equilibrated with the Tris buffer. Proteins were eluted with a linear gradient of 0.1–0.8 M NaCl in the Tris buffer. Fractions containing ST-CS were pooled, dialyzed against the Tris buffer, and then subjected to an anion exchange chromatography using a tandem of three HiTrapQ HP columns equilibrated with the Tris buffer. Proteins were eluted with a linear gradient of 0–1.0 M NaCl in the Tris buffer. Fractions containing ST-CS were pooled and concentrated with Centriprep-30 (Amicon).

### 2.2. Crystallization

Crystals of ST1805-CS and ST0587-CS were obtained by the hanging-drop vapor-diffusion method at 20°C. For crystallization of ST1805-CS, a drop (2 *μ*L) of protein solution (5 mg/mL) was mixed with 1 *μ*L of a reservoir solution containing 2 M ammonium sulfate, 0.1 M HEPES pH 7.0, and 2% (w/v) PEG400 and the mixture solution was equilibrated against 0.5 mL of the reservoir solution. Pillar-shaped crystals grew into a size of 0.2 × 0.1 × 0.3 mm within a week.

Crystals of ST0587-CS were obtained under a similar condition. From a mixture of 2 *μ*L of protein solution (1 mg/mL) and 1 *μ*L of reservoir solution comprising 3 M sodium formate and 0.1 M HEPES pH 7.0, bipyramidal crystals of ST0587-CS grew into a size of 0.2 × 0.2 × 0.2 mm within a week.

### 2.3. Diffraction Data Collection

Crystals of both were soaked for 10 min in the reservoir solution containing cryoprotectant (30% (v/v) glycerol). Subsequently crystals were flash-cooled with liquid nitrogen. During the data collection, the crystal was kept at 100 K under a gas flow of cold nitrogen from a cryostream. Diffraction images were collected using a CCD detector (MarResearch) at the beamline BL44B2 of SPring-8 (Harima, Japan). Diffraction data were processed with Mosflm [16], SCALA [17], and TRUNCATE [18] incorporated in the CCP4 program suite [19].

### 2.4. Structural Analysis

The crystal structure of ST1805-CS was solved with CNS1.0 [20] by the molecular replacement method using a model of the open form of SS-CS (PDB ID: 1O7X) as an initial model. After rotation and translation searches, the rigid-body refinement was carried out over the resolution range 15–4 Å. A proper solution with Rfactor of 0.45 was given with space group P2_1_. Crystallographic refinements were executed by repeatedly applying simulated annealing, conjugated gradient minimization, and B-factor refinements with the resolution range of 14.8–2.0 Å. Manual model buildings were executed with XtalView [21].

The structure of ST0587-CS was determined in the same manner using the open form of* Thermus thermophilus* CS (PDB ID: 1IOM) as a search model. After rotation and translation searches, the rigid-body refinement was carried out over the resolution range 15–4 Å. A proper solution with Rfactor of 0.49 was given with space group P4_1_2_1_2. Crystallographic refinements were executed by repeatedly applying simulated annealing, conjugated gradient minimization, and B-factor refinements with the resolution range of 43.0–2.7 Å. The qualities of the final models were assessed by using the program PROCHECK [22]. The statistics for data collection and refinements were summarized in Table 1.

Coordinates and structural parameters had been published with Protein Data Bank under accession codes 1VGM for ST1805-CS and 1VGP for ST0587-CS.

Figures were generated with Clustal W [23], ESPript [24], Pymol [25], and LigPlot+ [26].

### 2.5. Citrate Synthase Assays

Citrate synthase activity was assayed as previously described [27]. The assay mixture (300 *μ*L) contained 0.2 mM oxaloacetate, 0.15 mM acetyl-CoA, and 0.2 mM 5,5′-dithiobis(2-nitrobenzoic acid) in a buffer composed of 20 mM sodium phosphate, pH 7.0, and 2 mM EDTA. The assay temperature was stated in the text.

The dependence of catalytic activity on temperature was studied by assaying the citrate synthase activity for three minutes in the temperature range 30–100°C. In this condition, the substrates and the dye were not destructed during the time course of the assay even above 100°C (data not shown).

Thermal inactivation studies were carried out by incubating the mixture solution at 95°C for given periods. Aliquots were removed at known intervals and rapidly cooled in ice. Remaining enzyme activity was measured at 70°C under the assay condition described above.

## 3. Results

### 3.1. Sequence Analysis

**Figure 1:**
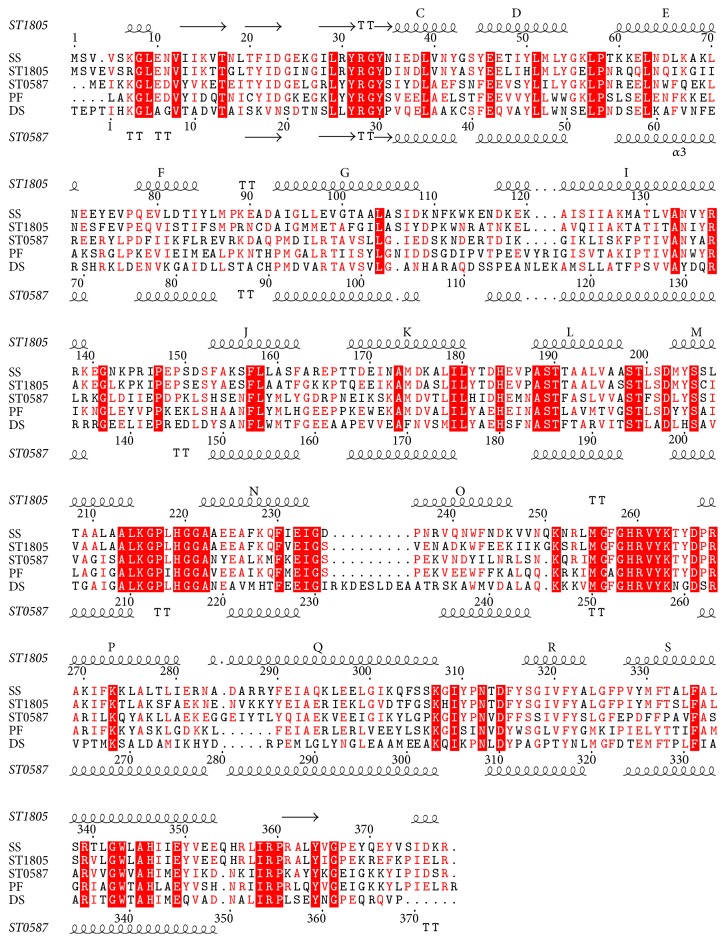
Multiple alignment of sequences for microbial CSs. SS; CS from* S. solfataricus*, ST1805; ST1805-CS, ST0587; ST0587-CS, PF; CS from* Pyrococcus furious*, DS; CS from* Arthrobacter Ds2-3R*. The secondary structure elements were indicated and labeled as C to G and I to S for *α*-helices, whose nomenclature was proposed by Remington et al. [8]. Identical residues were shaded in red; similar residues were colored red.

Figure 1 shows the sequence alignment among five CSs from a psychrophile and four hyperthermophiles. Four CSs from thermophiles show > 30% amino acid sequence identity with one another, whereas the sequence identities between them and CS from psychrophile* Arthrobacter Ds2-3R* (DS-CS) is much lower (18%). This suggests that this homology level is essential for expressing enzymatic activity of CS for all species. Two isozymes of ST-CS share 45% identity with each other. ST1805-CS is related closely to CS from the same genus* Sulfolobus solfataricus* (SS-CS), and they share 68% amino acid sequence identity. On the other hand, ST0587-CS exhibits 51% sequence identity with CS from hyperthermophilic archaeon* Pyrococcus furiosus* (PF-CS). All of these four CSs are thermophilic or hyperthermophilic enzymes, suggesting that a limited number of the amino acid residues contribute to thermostability for CSs or that distinct strategy for thermostability is employed between the two isozymes.

Table 2 shows the amino acid composition of these CSs. There is no correlation between growth temperature of the source organisms and the content of thermolabile residues asparagine and glutamine, deamidation of which causes irreversible inactivation. However, the content of two charged residues glutamate and lysine shows an increased tendency to develop thermotolerance as well as the cysteine and methionine content with decreased tendency [28, 29]. Charged residues would form ion pairs to enhance structural rigidity and therefore it should be considered in correlation with thermostability.

### 3.2. Temperature Dependence of Enzyme Activity

**Figure 2:**
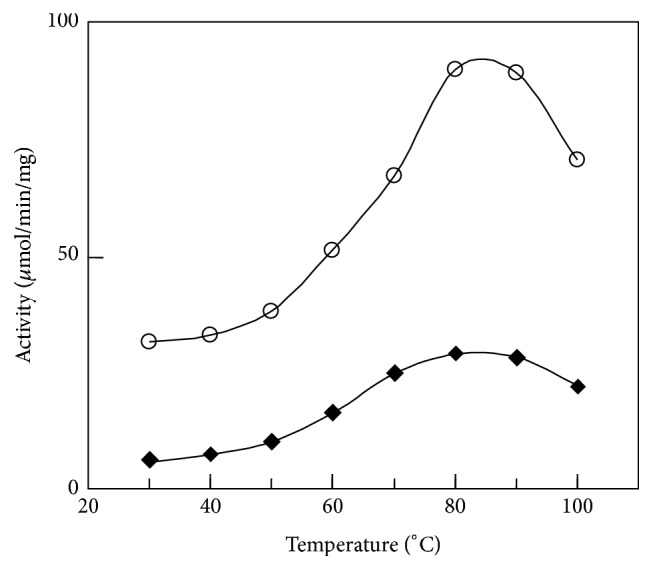
Temperature dependence of catalytic activity for ST1805-CS (open circle) and ST0587-CS (solid diamond), respectively.

Figure 2 shows temperature dependency of catalytic activity for ST-CSs. The temperature optima of catalytic activity for ST-CSs were determined by carrying out enzymatic assays over a time period of 1 min at different temperatures between 30 and 100°C. The duration for heat treatment is short enough to be stable without irreversible inactivation. ST1805-CS and ST0587-CS exhibit similar activity profiles and have similar optimal temperatures.

### 3.3. Thermostability

**Figure 3:**
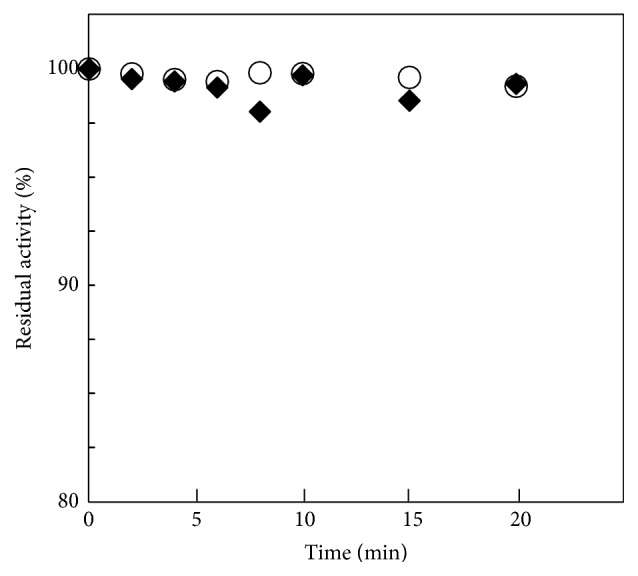
Time course of thermal inactivation of ST1805-CS (open circle) and ST0587-CS (solid diamond) at 95°C, respectively.

Figure 3 shows plots of thermal inactivation at 95°C for ST-CSs. The activity of two isozymes was almost unchanged at this temperature during the experience. However the stability of ST0587-CS was slightly lower than ST1805-CS; the activities of both ST-CSs were reduced only < 2% for preincubation for 5 min.

### 3.4. Structure of ST1805-CS at 2.0 Å Resolution

**Figure 4:**
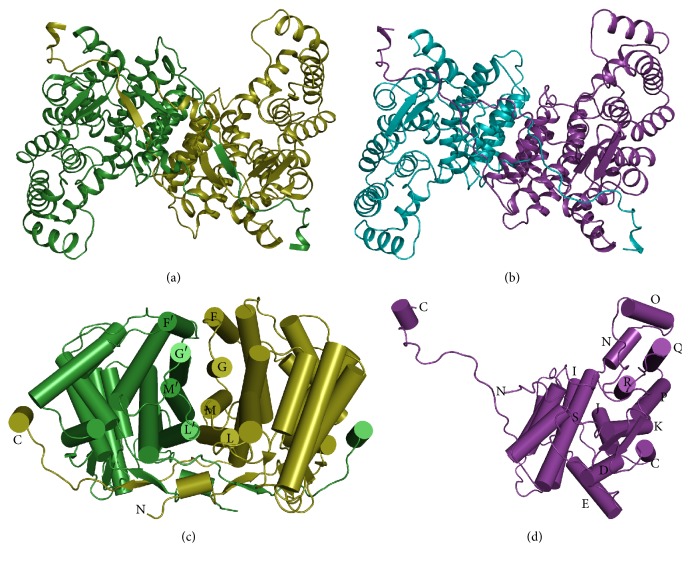
Overview of structures of homodimer of ST-CSs. The view is down a crystallographic 2-fold axis in the crystal of ST0587-CS, which relates the two subunits of the dimer. (a) Structure of ST1805-CS. Polypeptide chains A and B are colored in green and lime, respectively. (b) Structure of ST0587-CS. Each polypeptide is colored in cyan and purple, respectively. Polypeptide chains are shown with a ribbon model. (c) View of the dimer interface of ST1805-CS. (d) View of the monomer of ST0587-CS. Polypeptide chains are shown with a cylinder model.

ST1805-CS crystallized in a monoclinic crystal belonging to P2_1_ that diffracted X-rays up to 2.0 Å resolution. In this crystal, the asymmetric unit contains two monomers having nearly identical overall structures (Figure 4(a)). The final model is comprised of 376 amino acid residues per subunit (two residues in the N-terminus are disordered), 341 water molecules, a glycerol molecule, and a sulfate ion. In the dimeric structure, each subunit is subdivided into a large domain with 11 *α*-helices (C-G, I-M, and S helices, residues 3–216 and 325–378) and a small domain with five *α*-helices (N-R helices, residues 217–324) (Figure 4(d)). Four helices (F, G, M, and L helices) in the large domain confront the corresponding helices in the other subunit, forming a core helix bundle (F-F′, G-G′, M-M′, and L-L′ helices; the prime indicates the residue from the other subunit) (Figure 4(c)). The dimeric structure is further strengthened by an extended C-terminal loop, which involves a *β*-strand interacting with the first *β*-strand (residues 13–17) in the N-terminus of the other subunit. The second to the forth *β*-strands (residues 20–23, 28–31, and 34-35) in the N-terminus form an antiparallel *β*-sheet, capping the postulated active site.

The two peptides in the dimeric structure are superimposed with a root mean square (r.m.s.) deviation of 0.41 Å. A noticeable difference is seen preferentially in the small domain, that is, it is much larger (0.65 Å) than that for the large domains (0.23 Å). This difference can be explained by taking the protein packing into account. The small domain of subunit B extrudes into the open space in the unit cell, whereas the small domain of subunit A contacts with I helix and GI loop in the large domain of a neighboring dimer molecule maintaining the crystal structure. The asymmetric environments of the small domains cause a remarkable difference in the motional freedom of the small domains.

It is suggested that there is a hinge region between the two domains, around which the small domain is capable of undergoing a rigid-body rotation. One of such hinge regions is located at the loop between helices M and N, in which two glycine residues (Gly220 and Gly221) occur in succession. This pair of glycine residues in the loop structure is conserved among all CSs (Figure 1).

**Figure 5:**
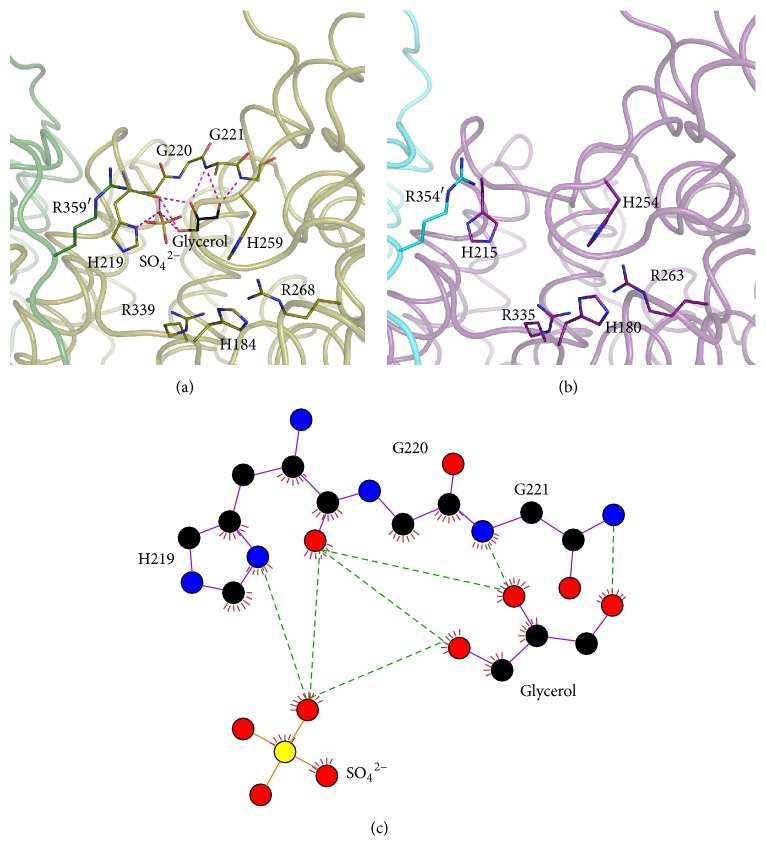
View in the active site of ST1805-CS (a) and ST0587-CS (b), respectively. Polypeptide chains are drawn in a ribbon model. (c) Schematic diagram of ligand binding mode in the active site of ST1805-CS. Oxygen, nitrogen, and sulfur atoms are shown in red, blue, and orange, respectively. Dotted lines indicate hydrogen bonds.

In the hinge region in subunit A of ST1805-CS, a glycerol molecule interacts with two glycine residues (Gly220, Gly 221) and interacts with His219 with a sulfate ion (Figure 5(a)). The latter residue is one of the putative substrate-binding residues involved in the binding of citrate and oxaloacetate in microorganism CSs [30]. His184, His259, Arg268, Arg339, and Arg359′, which are important residues for substrate binding, are exposed to the inner surface of the open cleft. Because these amino acids are also conserved in a wide variety of CS sequences, it is highly probable that ST1805-CS has same catalytic mechanism as proposed for other CSs.

In the structure of subunit B, some water molecules are found in the active site, accompanying hydrogen bonds with side chains of three arginine residues (Arg268, Arg339, and Arg359′) and one histidine residue (His184).

### 3.5. Structure of ST0587-CS at 2.7 Å Resolution

ST0587-CS crystallized into an orthorhombic crystal belonging to P4_1_2_1_2 that diffracted X-rays up to 2.7 Å resolution. In this crystal form, the asymmetric unit contains one monomer. This citrate synthase also forms a dimeric structure, the 2-fold axis of which coincides with the crystallographic 2-fold axis (Figure 4(b)). The final model is comprised of 373 amino acid residues and 40 water molecules. As observed for ST1807-CS, each subunit is subdivided into a large domain with 11 *α*-helices (C-G, I-M, and S helices, residues 1–212 and 321–373) and a small domain with five *α*-helices (N-R helices, residues 213–320) (Figure 4(d)). The principal architecture of the dimeric structure is the same as that found in ST1807-CS; namely, a core helix bundle is formed by four pairs of helices (F-F′, G-G′, M-M′, and L-L′ helices) and the C-terminal loop extends to the other subunit. The N-terminal region is folded into three strands (residues 18–21, 26–29, and 32-33), forming an antiparallel *β*-sheet, whereas an intersubunit *β*-sheet found in ST1805-CS is absent in ST0587-CS.

In the structure of ST0587-CS, six putative substrate-binding residues (His180, His215, His254, Arg263, Arg335, and Arg354′) were also structurally conserved (Figure 5(b)). Although the arrangement of these residues was almost identical to that of subunit A of ST1805-CS, no ligands but water molecules are identified in the active site due to the resolution limitation. These variations in the active site resulted in positional flexibility of the small domain relative to the large domain.

## 4. Discussion

The present results show that two genes obtained from* Sulfolobus tokodaii* encode isozymes that exhibit citrate synthase activity. It is known that many organisms possess more than one gene encoding citrate synthase [31–33]. The enzyme from eukarya, Gram-positive bacteria, and archaea shows homodimeric structure, whereas a Gram-negative bacterium* E. coli* has the other isozyme in hexameric form [34, 35].* E. coli* hexameric enzyme is citrate synthase as well as dimeric one from other organisms, while the other dimeric enzyme of* E. coli* is 2-methylcitrate synthase, whose substrates are propionyl-CoA and oxaloacetate, although it also has minor activity with acetyl-CoA [36, 37]. For another example,* Thermoplasma acidophilum* has two CS genes, Ta0169 and Ta0819, which share rather high sequence identity of 68%; thus they might show similar conformation and function similarly to each other [38]. Therefore we needed to express and purify recombinant proteins and investigate catalytic activity at the first step.

Both ST-CSs exhibit primary citrate synthase activity, which was slightly higher in ST1805-CS than ST0587-CS at high temperatures, and the dependency of catalytic activity on temperature is similar in both (Figures 2 and 3). This result is reasonable because we could find a tendency in their amino acid composition of their sequences of two isozymes toward increasing charged residues. Since charged residues might form ion pairs to develop their thermal adaptation mechanism, next we investigated the structures of two isozymes of ST-CSs and made detailed comparison between them to search candidate factors responsible for adaptation to high temperatures.

Comparison between overall structures of ST-CSs shows that the r.m.s. deviation between corresponding 373 C*α* atoms of each monomer is 1.27 Å. As the small domain increases flexibility compared with the large domain, we compared two domains separately. The rmsds from the large and the small domains showed only slight difference of 1.25 Å and 1.13 Å, respectively. It indicated that the whole dimer conformations are highly conserved between the open forms of ST-CSs. The relative positions of the large and the small domains also resemble each other.

**Figure 6:**
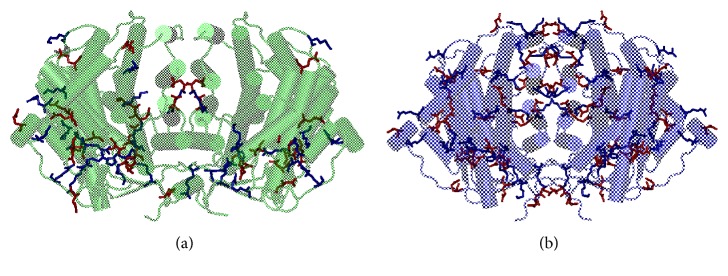
Charged residues forming ion pairs in the dimer of ST1805-CS (a) and ST0587-CS (b). Polypeptide chains are represented as a cylinder model. The positively and negatively charged residues are drawn as a line model and colored blue and red, respectively.

Since catalytic activities of both ST-CSs show similar dependency on temperature, we searched common structural features in terms of the electrostatic interactions. Unexpectedly, the structures of two isozymes exhibit that the numbers and the distributions of the ion pairs are quite different between ST-CSs (Figure 6). The numbers of ion pairs composed of residues of opposite charge situated within 4.0 Å were 36 and 55 in each dimer of ST1805-CS and ST0587-CS, respectively. The ion pairs were more dispersed throughout the dimer surface of ST0587-CS than ST1805-CS.

**Figure 7:**
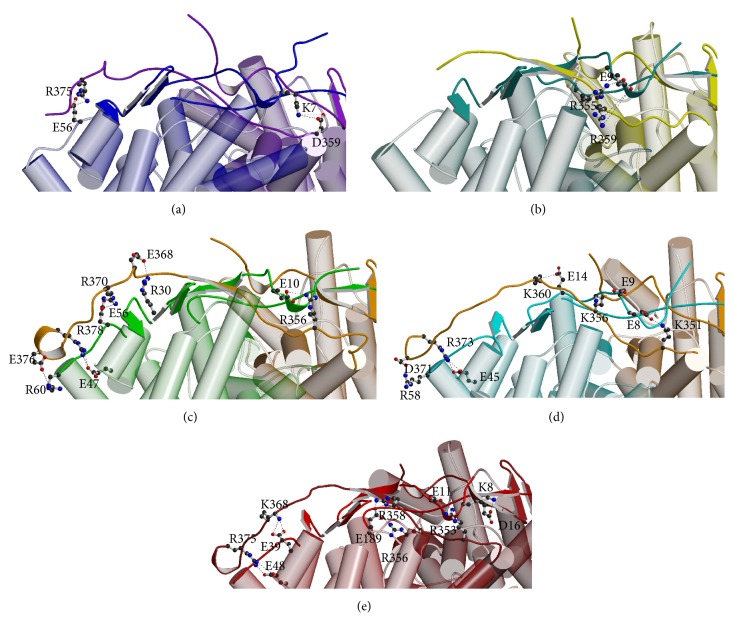
Ion pairs and networks in the terminal domain of PF (a), SS-CS (b), ST1805-CS (c), ST0587-CS (d), and PF-CS (e). Polypeptide chains are shown with a cylinder model. Residues involved in ion pairs are drawn as a ball-and-stick model. Carbon, oxygen, and nitrogen atoms are shown in black, red, and blue, respectively. Hydrogen bonds are indicated as a dotted line.

In particular, we found that the same numbers of the ion pairs are located in the C-terminal region between monomers in both ST-CSs. Arginine residue Arg378/373 (ST1805-CS/ST0587-CS) in the most C-terminal end was salt-bridged to a glutamate Glu47′/45′ in D′ helix, and the third C-terminal Glu376/Asp371′ interacted with Arg60′/58′ in E′ helix of neighboring subunit as well (Figure 7). Another three ion pairs were formed by different residues between two isozymes. In ST1805-CS, three arginine-glutamate pairs Arg370-Glu56′, Glu368-Arg30′, and Arg356-Glu10′ are formed, of which the new *β*-strand made across both termini from each subunit lies between the former two pairs and the latter one. On the other hand, these three interactions of ST0587-CS were modified as lysine-glutamate pairs, that is, Lys360-Glu14′, Lys356-Asp9′, and Lys351-Glu8′. These common ion pair interactions within the C-terminal loop increased to probably stabilize the formation of the active site. The number of this ion pairs localized there and the thermal stability and activity seem to correlate with each other (Figure 7). This trend is in line with previous result of a deletion mutation experience of PF-CS, in which two- and 13-residue deletion in the C-terminal end of Pf-CS showed that the shorter the C-terminal end becomes, the lower the temperature optimum for catalytic activity gets down even though 13-residue deletion mutant shows being more thermostable than two-residue deletion mutant [39]. In SS-CS, terminal region shows disorder and the C-terminal five residues are missing, but almost all residues responsible for electrostatic interactions within this region shown in ST1805-CS are conserved in SS-CS.

**Figure 8:**
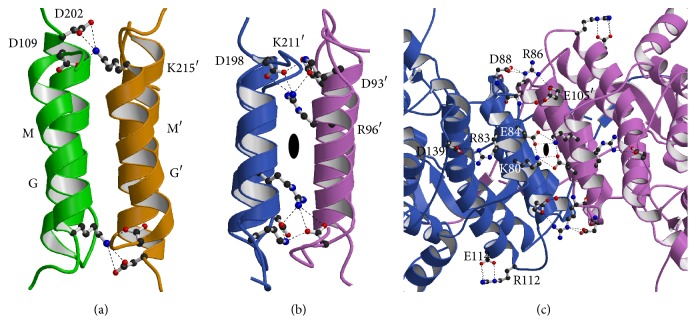
(c) Ion pairs and networks at the molecular surface. (a) Ion pairs near core helices G and M in ST1805-CS. (b) Ion pairs between core helices G and M in ST0587-CS. (c) Extensive ion pair networks found on the surface perpendicular to the axis of the central helix bundle of ST0587-CS. The view is opposite side to that of Figure 2. The residues involved in the ion pairs were drawn as a ball-and-stick model. Polypeptide chains are represented as a ribbon model. Carbon, oxygen, and nitrogen atoms are shown in black, red, and blue, respectively. Hydrogen bonds are indicated as a dotted line.

Next, we found intensive electric interactions at the molecular surface (Figure 8). In ST1805-CS, an ion pair network was comprised of three residues; Asp109 in GI loop and Asp202 in LM loop were salt-bridged with Lys215′ in M′N′ loop of neighboring subunit (Figure 8(a)). In ST0587-CS, an ion pair network was organized by four residues, Asp93 and Arg96 in G helix, Lys211 in MN loop, and Asp198′ in L′M′ loop of neighboring subunit, and continued into hydrophobic center of the helix bundle (Figure 8(b)). Although the trend in which the number of residues comprising the ion pair network is higher than mesophilic CS and lower than hyperthermophilic CS is found in this region of both ST-CSs [40–43], the location is different in ST1805-CS at the loop region. In addition, ST0587-CS has several ion pairs at the surface including intersubunit regions (Figure 8(c)). Because it is difficult to estimate the contribution of the ion pair network at the molecular surface under the physiological low-pH condition for ST-CSs, these ion pairs should be excluded for adaptation mechanism for high temperatures in present study.

## 5. Conclusion

In this study, we investigated two isozymes of CS from* Sulfolobus tokodaii* strain 7 using crystallographic and biochemical techniques. These isozymes show similar high catalytic activity and stability at high temperatures and then common structural features of adaptation mechanism for high temperatures were searched. Structural comparison between them showed large differences in the number of ion pairs, but identical numbers of ion pairs located in the terminal regions were found in both ST-CSs. We propose that these terminal interactions are essential for adaptation mechanism to high temperatures in CS from thermophilic microorganisms.

## Figures and Tables

**Table 1:** Data collection and final refinement statistics.

	ST1805-CS	ST0587-CS
Data collection		
Wavelength (Å)	1.0	1.0
Resolution (Å)	30.3–2.0	54.2–2.7
Data completion (%) (outer shell)	95.4 (97.1)	99.8 (99.9)
Number of unique reflections	57694	22985
Multiplicity	3.6	7.1
*R*_sym_^1^ (%) (outer shell)	8.0 (19.8)	9.7 (47.0)
Space group	P2_1_	P4_1_2_1_2
Unit cell (Å)	*a* = 62.87	*a* = 121.60
	*b* = 94.17	*b* = 121.60
	*c* = 77.62	*c* = 108.83
	*α* = *γ* = 90°	*α* = *β* = *γ* = 90°
	*β* = 97.22°	
Refinement		
Resolution limit (Å)	14.79–2.3	43.0–2.7
Number of protein atoms	5994	3044
Number of water molecules	347	40
*R*_work_^2^ (%) (outer shell)	19.1	21.7
*R*_free_ (%) (outer shell)	23.0	24.6
r.m.s. deviation of bond length (Å)	0.006	0.007
r.m.s. deviation of bond angle (°)	0.70	1.00
Estimated error from Luzzati plot (Å)	0.21	0.33

^1^ *R*_sym_ = ∑_hkl_∑_*i*_|*I*_*i*_ − 〈*I*〉|/∑_hkl_∑_*i*_ *I*_*i*_, where *I*_*i*_ is the intensity of an individual reflection and 〈*I*〉 is the mean intensity obtained from multiple observations of symmetry related reflections.

^2^ *R*_work_ = ∑_hkl_(*F*_obs_ − |*F*_calc_|)/∑_hkl_|*F*_obs_| (9.2% randomly omitted reflections were used for calculation of *R*_free_).

**Table 2:** Total amino acid composition.

	SS		ST1805		ST0587		PF		DS	
Gly	21	(5.6)	24	(6.3)	25	(6.7)	29	(7.6)	28	(6.6)
Ala	37	(9.8)	35	(9.3)	22	(5.9)	28	(7.3)	37	(8.7)
Val	21	(5.6)	18	(4.8)	19	(5.1)	21	(5.5)	19	(4.5)
Leu	39	(10.3)	31	(8.2)	35	(9.4)	37	(9.7)	35	(8.2)
Ile	27	(7.2)	37	(9.8)	37	(9.9)	34	(8.9)	28	(6.6)
Phe	17	(4.5)	18	(4.8)	18	(4.8)	10	(2.6)	23	(5.4)
Tyr	22	(5.8)	21	(5.6)	27	(7.2)	26	(6.8)	16	(3.8)
Trp	3	(0.8)	3	(0.8)	2	(0.5)	7	(1.8)	3	(0.7)
Cys	0	(0.0)	2	(0.5)	0	(0.0)	1	(0.3)	7	(1.6)
Met	8	(2.1)	9	(2.4)	8	(2.1)	9	(2.4)	18	(4.2)
Ser	19	(5.0)	23	(6.1)	23	(6.2)	19	(5.0)	25	(5.9)
Thr	19	(5.0)	20	(5.3)	10	(2.7)	16	(4.2)	28	(6.6)
Asn	18	(4.8)	15	(4.0)	14	(3.8)	12	(3.1)	17	(4.0)
Gln	8	(2.1)	8	(2.1)	5	(1.3)	5	(1.3)	8	(1.9)
Asp	18	(4.8)	12	(3.2)	21	(5.6)	11	(2.9)	27	(6.3)
Glu	31	(8.2)	34	(9.0)	32	(8.6)	40	(10.5)	26	(6.1)
His	5	(1.3)	7	(1.9)	6	(1.6)	8	(2.1)	15	(3.5)
Lys	31	(8.2)	28	(7.4)	33	(8.8)	34	(8.9)	23	(5.4)
Arg	18	(4.8)	17	(4.5)	20	(5.4)	17	(4.5)	24	(5.6)
Pro	15	(4.0)	16	(4.2)	16	(4.3)	17	(4.5)	19	(4.5)

Total	377		378		373		381		426	
